# Optimized Methods to Quantify Tumor Treating Fields (TTFields)-Induced Permeabilization of Glioblastoma Cell Membranes

**DOI:** 10.3390/mps8010010

**Published:** 2025-01-22

**Authors:** Melisa Martinez-Paniagua, Sabbir Khan, Nikita W. Henning, Sri Vaishnavi Konagalla, Chirag B. Patel

**Affiliations:** 1 Department of Neuro-Oncology, The University of Texas MD Anderson Cancer Center, 1515 Holcombe Blvd., Unit 1002, BSRB S5.8116b, Houston, TX 77030, USA; mmartinez33@mdanderson.org (M.M.-P.); skhan15@mdanderson.org (S.K.); nwhenning@mdanderson.org (N.W.H.);; 2 Cancer Biology Program, The University of Texas MD Anderson Cancer Center/The University of Texas Health Science Center at Houston Graduate School of Biomedical Sciences, Houston, TX 77030, USA; 3 Neuroscience Graduate Program, The University of Texas MD Anderson Cancer Center/The University of Texas Health Science Center at Houston Graduate School of Biomedical Sciences, Houston, TX 77030, USA

**Keywords:** cell membrane permeability, flow cytometry, lactate dehydrogenase (LDH), method optimization, tumor treating fields (TTFields)

## Abstract

Glioblastoma (GBM) is a lethal primary brain cancer with a 5.6% five-year survival rate. Tumor treating fields (TTFields) are alternating low-intensity electric fields that have demonstrated a GBM patient survival benefit. We previously reported that 0.5–24 h of TTFields exposure resulted in an increased uptake of FITC-dextran fluorescent probes (4–20 kDa) in human GBM cells. However, this approach, in which a fluorescence plate-based detector is used to evaluate cells attached to glass coverslips, cannot distinguish FITC-dextran uptake in live vs. dead cells. The goal of the study was to report the optimization and validation of two independent methods to quantify human GBM cell membrane permeabilization induced by TTFields exposure. First, we optimized flow cytometry by measuring mean fluorescence intensity at 72 h for 4 kDa (TTFields 6726 ± 958.0 vs. no-TTFields 5093 ± 239.7, *p* = 0.016) and 20 kDa (7087 ± 1137 vs. 5055 ± 897.8, *p* = 0.031) probes. Second, we measured the ratio of lactate dehydrogenase (LDH) to cell viability (measured using the CellTiter-Glo [CTG] viability assay); the LDH/CTG ratio was higher under TTFields (1.47 ± 0.15) than no-TTFields (1.08 ± 0.08) conditions, *p* < 0.0001. The findings using these two independent methods reproducibly demonstrated their utility for time-dependent evaluations. We also showed that these methods can be used to relate the cell membrane-permeabilizing effects of the non-ionizing radiation of TTFields to that of an established cell membrane permeabilizer, the non-ionic detergent Triton-X-100. Evaluating carboplatin ± TTFields, the LDH/CTG ratio was significantly higher in the TTFields vs. no-TTFields condition at each carboplatin concentration (0–30 µM), *p =* 0.014. We successfully optimized and validated two cost-effective methods to reproducibly quantify TTFields-induced human GBM cancer cell membrane permeabilization.

## 1. Introduction

Glioblastoma (GBM) is a lethal primary brain cancer, with a 5-year survival rate of 5.6% despite surgery, radiation therapy, and temozolomide chemotherapy. The most recent U.S. Food and Drug Administration-approved therapy for patients with GBM is tumor treating fields (TTFields), alternating low-intensity electric fields that slow tumor growth and disease progression. As a monotherapy, 200 kHz TTFields are non-inferior to physicians’ best choice of chemotherapy in the recurrent disease setting [1]. In combination with adjuvant monthly temozolomide, TTFields were shown to prolong patients’ median overall survival duration by 4.9 months and more than double the 5-year survival rate to 13% compared to adjuvant monthly temozolomide alone in newly diagnosed GBM patients [2,3]. The survival benefits of 200 kHz TTFields combined with temozolomide chemotherapy, versus temozolomide alone, have also been demonstrated in the post-marketing, real-world setting in patients with recurrent [4] and newly diagnosed [5,6] GBM. TTFields have also been approved for patients with malignant pleural mesothelioma [7] and non-small cell lung cancer [8]. TTFields are delivered in patients via adhesive electrode transducer arrays attached to the skin surrounding the target organ (e.g., brain or lungs) [9]. The most common side effect of TTFields therapy is skin toxicity, which is preventable in most cases [10,11,12], and if it occurs, it can be managed with slight adjustments to the placement of the electrode arrays and/or topical medications [13].

The first recognized anti-cancer biological activity of TTFields was their anti-mitotic effect [14]; they also disrupt cell membrane integrity [15,16] and increase blood–brain barrier permeability, thereby presumably improving systemic therapy access to the central nervous system [17,18]. Various biological mechanisms and effects of TTFields, including permeabilization of cell membranes, are under investigation. Using scanning electron microscopy and fluorescence-based readouts, we previously showed that they induce the formation of pores in the human GBM cell membrane [19] in a manner resembling that of reversible electroporation [20]. We previously reported an increased uptake of FITC-dextran fluorescent probes in U87 human GBM cells using a fluorescence plate-based detector for FITC-dextrans (4–20 kDa) after TTFields exposure, when cells were attached to a glass coverslip (GCS) [19]. However, this approach cannot distinguish between FITC-dextran uptake in live vs. dead cells. With increasing clinical applications of TTFields, it is important to identify methods that can reliably and accurately detect the cell membrane-permeabilizing effects of TTFields.

Flow cytometry (FC) is a single-cell technology that measures cell-specific scatter and fluorescence properties. FC is used in many areas of science, in particular, biotechnology and medicine. For example, FC is used in immunological studies to identify multiple phenotypic subsets from a mixture of cells and to identify and isolate specific cells via cell sorting [21]. FC can also be used in many other cell biology readouts to evaluate processes such as apoptosis, necrosis, and ferroptosis [22].

Lactate dehydrogenase (LDH) is a stable enzyme found in all cells; it is released into the surrounding media when the cell’s plasma membrane is damaged under various experimental and pathological conditions, thereby leading to increased cell membrane permeability [23]. LDH release is a widely used method for evaluating cellular damage and insults in tissues and cells, as well as clinically assessing cell damage in the heart, liver, skeletal muscle, and blood [23,24].

In this study, we optimized and validated two cost-effective methods (FC- and LDH-based) to confirm our previous plate-based results in human GBM cells that were exposed or unexposed to TTFields. We performed a live cell-by-cell analysis and a membrane permeability assay to determine the uptake of FITC-dextran and LDH release to assess the permeabilization of the cell membrane in the presence or absence of TTFields exposure using optimized methods. We then used a normalized LDH/CellTiter-Glo (CTG) ratio to compare differences between TTFields-exposed and -unexposed cells.

## 2. Materials and Methods

### 2.1. Cell Culture and Reagents

The human GBM cell line U87-MG was obtained from the ATCC (Rockville, MD, USA) and grown in Dulbecco’s modified Eagle’s medium (DMEM) with high glucose, 1× antibiotic–antimycotic (Corning, Glendale, AZ, USA), and 10% fetal bovine serum ([FBS] MilliporeSigma, Burlington, MA, USA). The probes fluorescein isothiocyanate (FITC)-dextran FD4, FD20, and Triton-X-100 were purchased from MilliporeSigma. The live and dead dye propidium iodide was from BD (Franklin Lakes, NJ, USA). The CellTiter-Glo and LDH Glo kits were purchased from Promega (Madison, WI, USA). Carboplatin was purchased from SelleckChem (Houston, TX, USA).

### 2.2. GCS Preparation for the TTFields Experiment

**Figure 1:**
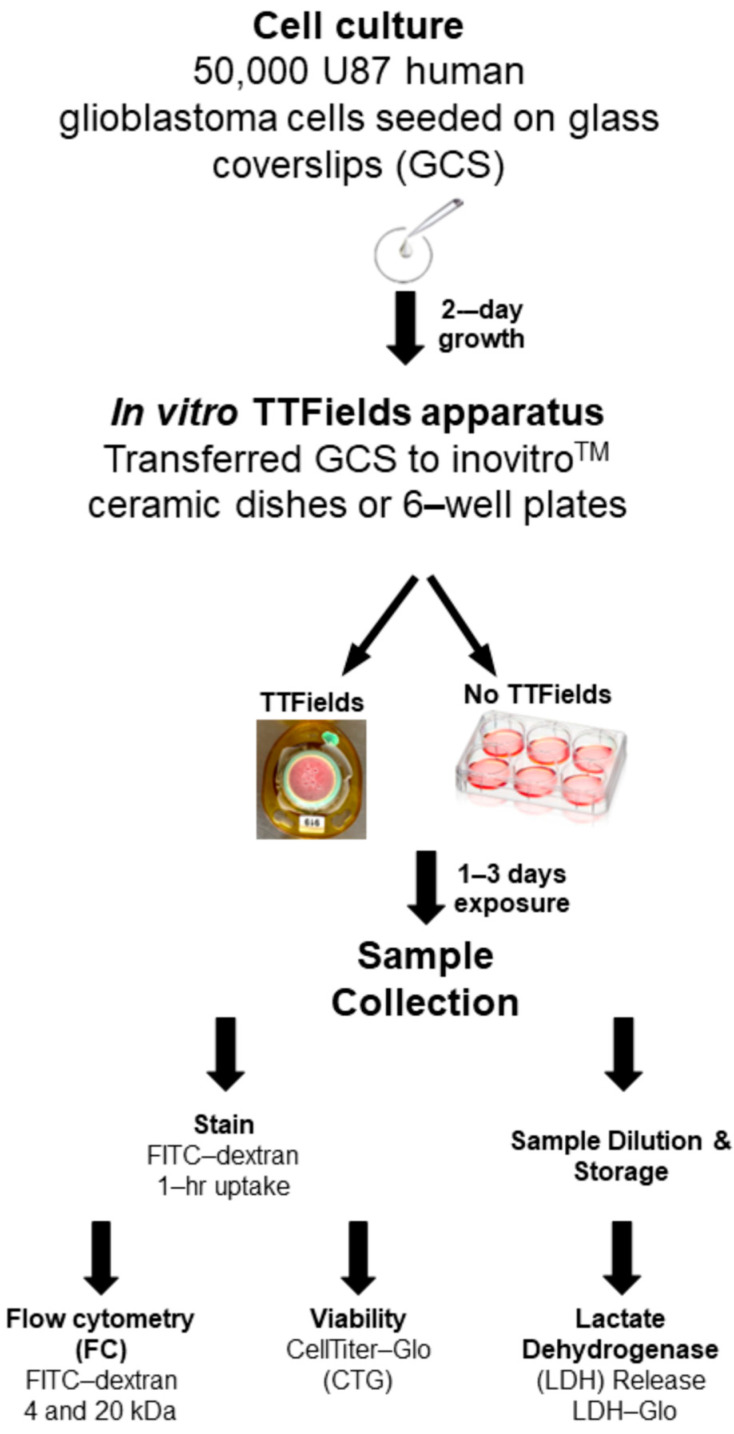
Overview of the experimental setup for the in vitro TTFields experiment and methods for readouts.

The cells were trypsinized, counted, and seeded at a density of 50,000 cells in 200 µL of DMEM/10% FBS/1× antibiotic–antimycotic on a 22 mm diameter GCS (Fisherbrand™ circular cover glasses, Thermo Fisher Scientific, Waltham, MA, USA) in a six-well plate. The cells were then incubated overnight at 37 °C and 5% CO_2_. The plates were inspected and filled with 2.5 mL of DMEM/10% FBS/1× antibiotic–antimycotic the following day, and the cells were left to grow for 24 h to ensure that they were in the growth phase. After 48 h of seeding, the GCSs were transferred to a ceramic dish of an inovitro™ in vitro TTFields apparatus (Novocure, Inc., Haifa, Israel). An overview of the experimental setup for the in vitro TTFields experiment and methods for the various readouts are summarized in Figure 1.

### 2.3. In Vitro TTFields Apparatus

The ceramic dishes with GCSs were placed on inovitro™ base plates (Novocure Ltd., Haifa, Israel), and 200 kHz TTFields (1–4 V/cm, peak to peak) were applied using an inovitro™ power generator. A target temperature of 37 °C in the ceramic dishes was maintained when TTFields were applied. The TTFields exposure lasted 24–72 h, after which the GCSs were removed and processed for the appropriate bioassays (details below). The ceramic dishes were covered with Parafilm to minimize evaporation. Corresponding control experiments (without TTFields) were conducted by placing equivalent GCSs in a conventional humidified tissue culture incubator (37 °C, 5% CO_2_) and growing cells in parallel with the TTFields-exposed GCSs. In separate experiments, TTFields (or no-TTFields) were combined with carboplatin chemotherapy (0–30 µM), which has poor blood–brain-barrier penetration. For the experiments involving Triton-X-100 or carboplatin, “control” refers to the conditions without chemical or drug exposure, respectively. Unless otherwise stated, each experiment was conducted at least twice, with four replicates for each condition and time point.

### 2.4. Flow Cytometry

We previously showed that after exposure to TTFields (1–4 V/cm, 200 kHz, 0.5–24 h duration), U87 human GBM cells increased the internalization of the 4 and 20 kDa FITC-dextrans, as measured using a plate-based detector when the cells were still attached to the GCS [19]. The feasibility of FITC-dextran probe uptake detection by flow cytometry (FC) was evaluated. We performed a live cell-by-cell analysis and membrane permeability assay to determine the uptake of FITC-dextran in the presence or absence of TTFields using optimized FC methods. The first approach was to evaluate the internalization of the FITC-dextran probe in a cell-per-cell manner using FC. The TTFields-exposed and -unexposed cells were detached from their GCSs using 400 µL of TrypLE™ solution. To halt the effects of trypsinization, 1.5 mL of DMEM media containing 10% FBS was added, and the cell suspension was collected and washed twice with 2 mL of Hanks’ Balanced Salt Solution per tube, spinning at 350× *g* for 5 min at 4 °C. The cells were stained using 500 µL of the appropriate FITC-dextran reagent, 4 or 20 kDa, and incubated in the dark for 1 h at 37 °C and 5% CO_2_. Later, the cells were washed twice with Hanks’ Balanced Salt Solution, resuspended in 150–200 µL of fluorescence-activated cell sorting buffer, and analyzed using Gallios FC (Beckman Coulter, Brea, CA, USA) equipment. Dead cell exclusion was performed using the live and dead dye PI. The results were analyzed using FlowJo software version 10.9.0 (Becton, Dickinson and Company, Ashland, OR, USA).

### 2.5. Cell Viability Measurement by CellTiter-Glo^®^ Assay

Cell viability or proliferation was evaluated using a luminescence-based cell viability assay (CellTiter-Glo^®^ [CTG], Promega) according to the manufacturer’s protocol. The cells on the GCS were cultured and exposed to TTFields using the inovitro™ system with a standard protocol for the indicated frequencies and durations in Section 2.3. CTG was performed by transferring GCS into six-well plates with 1 mL of culture media in each well and then adding 1 mL of CTG reagent (1:1 ratio). The plates were covered with foil and slowly shaken on the rocker to mix the content for 20–30 min. Next, 200 µL of the contents were transferred into the wells of a white 96-well plate, in triplicate from each experimental condition. The reaction mixture with culture media but no cells was used as background reading for each plate. Luminescence was measured using the 96-well plate reader (BioTek Synergy HTX Multimode Reader, Agilent, Santa Clara, CA, USA). The relative cell viability was calculated using no-TTFields (unexposed to TTFields) or control (unexposed to Triton-X-100 or unexposed to carboplatin) cells for each plate.

### 2.6. LDH Release Assay

Lactate dehydrogenase (LDH) release in cell culture media was evaluated using a luminescence-based LDH release assay (LDH-Glo™ Cytotoxicity Assay, #J2381, Promega, Fitchburg, WI, USA) according to the manufacturer’s protocol. The cells on the GCS were cultured and exposed to TTFields using the inovitro^TM^ system with a standard protocol [19] for the frequencies and durations mentioned in Section 2.3. At the end of the experiment, 10 µL of culture media were collected from each sample and diluted with 990 µL of LDH storage buffer (1:100) containing 200 mM Tris hydrochloride (pH 7.3), 10% glycerol, and 1% bovine serum albumin. The diluted samples were stored in a −80 °C freezer until analysis. The LDH estimation was performed using 50 µL of diluted (1:100) cell media samples and 50 µL of LDH assay detection reagents (containing lactate, nicotinamide adenine dinucleotide, reductase substrate, and Ultra-Glo™ recombinant Luciferase) in a white 96-well plate in duplicate. A standard curve with recombinant LDH (rLDH, 0–32 mUnit/mL) was also run in the same plates to quantitatively estimate the LDH in the samples. The plates were covered with foil and slowly shaken on the rocker to mix the content for 60 min. The luminescence was measured using the 96-well plate reader (BioTek Synergy HTX Multimode Reader). The absolute LDH release was calculated for the various conditions, i.e., TTFields or no-TTFields; Triton-X-100 or control (unexposed to Triton-X-100). The ratio of absolute LDH release to normalized (TTFields to no-TTFields; or Triton-X-100 to no Triton-X-100) CTG was calculated for each replicate.

### 2.7. Triton-X-100 Concentration Optimization by Light Microscopy

The concentration of Triton-X-100, a known non-ionic detergent, was optimized for use as a positive control. A total of 0.3–0.5 million U87 cells were seeded and allowed to attach for 24 h and then exposed to a range of Triton-X-100 concentrations (0–0.2% *v*/*v* in water) for 15 min in 6-well plates at 37 °C. The aim was to identify the optimal concentration of Triton-X-100 that did not substantially damage the cell membranes to the point of cell death, but that still induced membrane permeability. The optimal concentration was determined by capturing images under a light microscope (10× objective lens) in culture media, after washing out the Triton-X-100. The maximum concentration that did not cause cell detachment was considered to be the optimal concentration for further experiments.

### 2.8. Data and Statistical Analyses

Normally distributed data are presented as means ± standard deviation, and non-normally distributed data are presented as medians (interquartile range). Data are representative of at least two independent experiments unless otherwise noted. The statistical analyses were performed using GraphPad Prism (version 10.2) statistical software (Boston, MA, USA). Data were tested for normality using the Shapiro–Wilk test (a *p*-value < 0.05 indicated that the data were not normally distributed). Normally distributed data were analyzed using Student’s *t*-test (two-group comparisons) or an analysis of variance (ANOVA, more-than-two-group comparisons); statistically significant results from these parametric tests are denoted by the pound sign (#). Non-normally distributed data were analyzed using the Mann–Whitney U-test (two-group comparisons) or the Kruskal–Wallis H test (more-than-two-group comparisons); statistically significant results from these non-parametric tests are denoted by an asterisk (*). The significance level (α) was set at 0.05. For multiple two-group comparisons, α was adjusted using Bonferroni correction.

## 3. Results

### 3.1. Identifying the Optimal FITC-Dextran Concentration for the FC-Based Approach (Titration Range)

**Figure 2:**
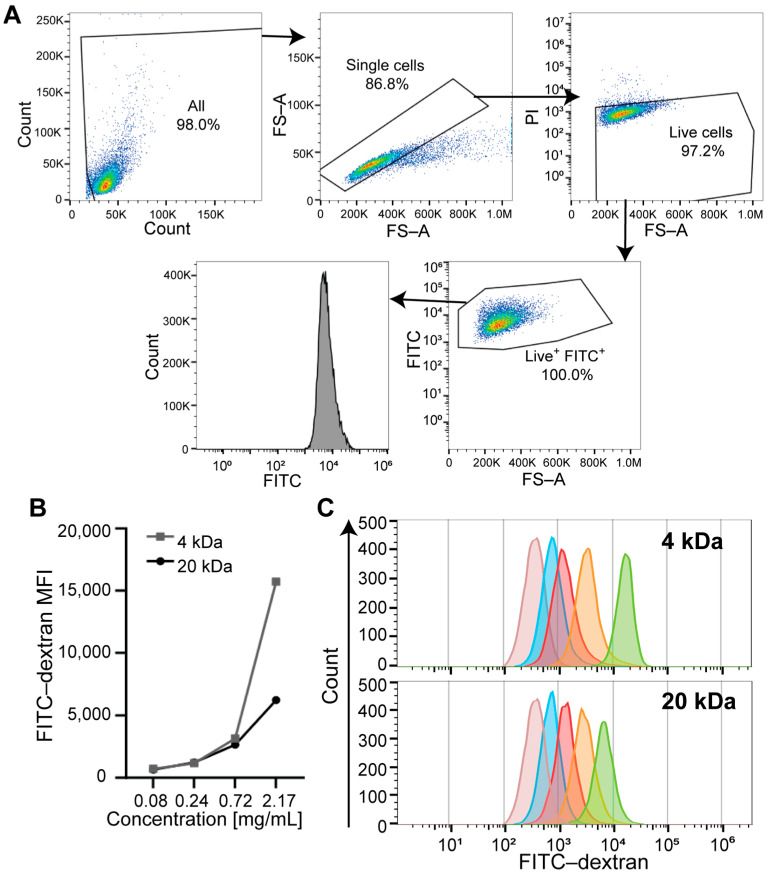
Determination of fluorescence probe concentration for FC. (**A**) Representative FC plots display the gating strategy to identify FITC-dextran-positive cells. U87 cells were incubated with FITC-dextran 4 or 20 kDa for 1 h, and 10,000 live FITC-positive cell events were recorded using FC. (**B**) The line plots show the MFI of the various concentrations of the FITC-dextran 4 or 20 kDa probes. The optimal concentration for each probe was determined to be 0.72 mg/mL. (**C**) The representative FC count histograms of the FITC-dextran probes at different concentrations. FITC = fluorescein isothiocyanate; FS = forward scatter; PI = propidium iodide.

To evaluate the minimum concentration (for cost-effectiveness) of FITC-dextran probe detection by FC, we first determined the optimal concentration in the U87 cells. The cells were exposed to different concentrations of FITC-dextran 4 or 20 kDa (0.08–2.17 mg/mL) for 1 h and then analyzed to determine the medium fluorescence intensity (MFI) on live cells, as shown in Figure 2A (gating strategy). We observed an increase in FITC-dextran 4 and 20 kDa dose-dependent internalization in U87 cells. On the basis of these initial titrations, we decided to use 0.72 mg/mL concentration for each of the two probes in all subsequent flow experiments to evaluate TTFields-induced cell membrane permeabilization (Figure 2B,C). The value of 0.72 mg/mL was chosen as the optimal concentration for each probe size because it was the minimal concentration that yielded measurable MFI as compared to unstained cells (good signal-to-background ratio).

### 3.2. TTFields Exposure Significantly Increases the Uptake of Different-Sized FITC-Dextran Probes (4 and 20 kDa) in U87 Human GBM Cells

In regard to the optimal Triton-X-100 concentration to serve as a positive control for cell membrane permeabilization, we observed that high concentrations (>0.05% *v*/*v* of Triton-X-100 in water) detached and killed the cells (Supplementary Figure S1), and lower concentrations (<0.025% *v*/*v*) did not affect the cells’ adhesion to the surface of the plate.

**Figure 3:**
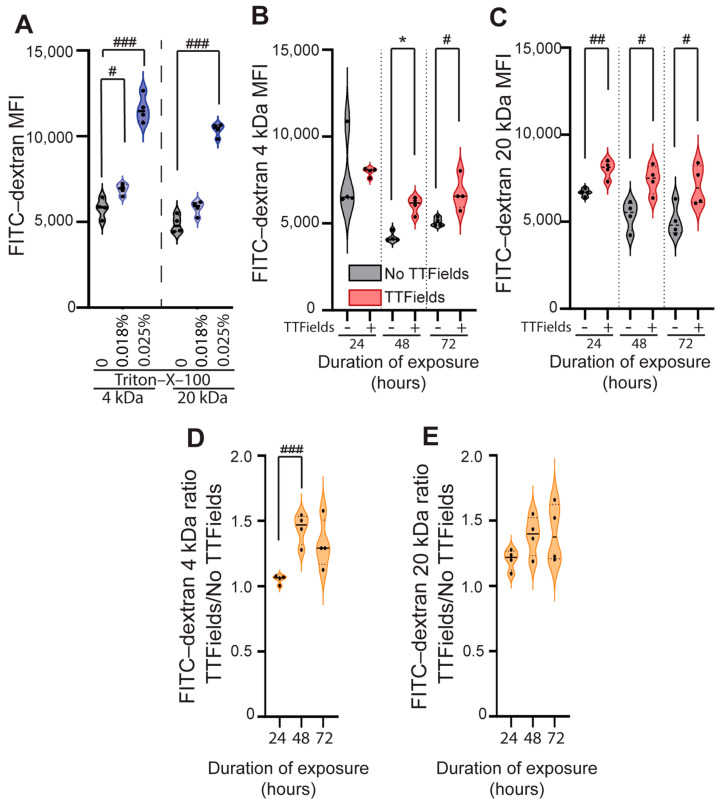
TTFields exposure (24–72 h) significantly increased the uptake of different-sized FITC-dextran probes (4 and 20 kDa). (**A**) Representative plots of the MFI of FITC-dextran probe uptake after a 10 min Triton-X-100 exposure. MFI of the (**B**) 4 kDa and (**C**) 20 kDa FITC-dextran probe uptake after TTFields exposure for 24–72 h. Normalized ratio of the FITC-dextran MFI in the TTFields-to-no-TTFields conditions for the (**D**) 4 kDa and (**E**) 20 kDa probes demonstrates that TTFields increased cell membrane permeability for the smaller 4 kDa probe, but not the larger 20 kDa probe, after a 48 h exposure. Three independent experiments were performed, and a parametric (#) or non-parametric test (*) was applied for statistical analysis. # or * indicates *p* < 0.05, ## *p* < 0.01, and ### *p* < 0.001 compared to the no-TTFields or control (unexposed) groups.

In separate experiments, we exposed the cells to 0.018% and 0.025% *v*/*v* solutions of Triton-X-100 in water for 10 min at 37 °C, incubated them for 1 h with the FITC-dextran probes (separate samples for 4 and 20 kDa), and measured the MFI by FC. There was a statistically significant increase in the uptake of fluorescent probes in a concentration-dependent manner for both probes: the 4 kDa probe (control = 5807 ± 565.5 vs. 0.018% *v*/*v* of Triton-X-100 = 6905 ± 327.1, *p* = 0.0152 [statistically significant after Bonferroni correction]; control = 5807 ± 565.5 vs. 0.025% *v*/*v* of Triton-X-100 = 11,596 ± 799.1, *p* < 0.0001 [statistically significant after Bonferroni correction]; Student’s *t*-test) (Figure 3A left) and the 20 kDa probe (control = 4872 ± 513.5 vs. 0.025% *v*/*v* of Triton-X-100 = 10,368 ± 376.6, *p* < 0.0001 [statistically significant after Bonferroni correction]; Student’s *t*-test) (Figure 3A right).

We observed a significant increase in the uptake of FITC-dextran 4 kDa in the TTFields-exposed cells at 48 h (TTFields 6184 [5545, 6451] vs. no-TTFields 4090 [4030, 4501], *p* = 0.028, Mann–Whitney U-test) and 72 h (TTFields 6726 ± 958.0 vs. no-TTFields 5093 ± 239.7, *p* = 0.016, Student’s *t*-test), but not at 24 h (Figure 3B). However, for the 20 kDa probe, there was a significant increase in uptake in the TTFields vs. no-TTFields conditions at all three time points using Student’s *t*-test (24 h: 802 ± 520.9 vs. 6678 ± 204.4, *p* = 0.003; 48 h: 7423 ± 817.9 vs. 5365 ± 830.2, *p* = 0.012; 72 h: 7087 ± 1137 vs. 5055 ± 897.8, *p* = 0.031) (Figure 3C).

Human GBM cell membrane permeabilization, as measured by the MFI of 4 kDa (Figure 3B) and 20 kDa (Figure 3C) for FITC-dextran probe uptake, was comparable between a 200 kHz TTFields exposure for 24–72 h and a 0.018–0.025% Triton-X-100 solution exposure for 10 min.

Next, the 4 kDa and 20 kDa FITC-dextran uptake over time was calculated as normalized MFI ratios under TTFields vs. no-TTFields conditions (Figure 3D,E); the only statistically significant increase in the MFI ratio over time occurred for the 4 kDa probe, at 48 h compared to 24 h (1.44 ± 0.12 vs. 1.05 ± 0.03; *p* = 0.0007 [statistically significant after Bonferroni correction] by Student’s *t*-test). This demonstrates that TTFields increased cell membrane permeability for small molecules (~4 kDa), but not larger molecules (~20 kDa), after a 48 h exposure. These results demonstrate that FC helps to detect permeabilization on the surface of the membrane due to TTFields exposure.

### 3.3. Optimized LDH Release Assay Detects TTFields-Induced Human GBM Cell Membrane Permeabilization in a Time-Dependent Manner, with High Sensitivity

**Figure 4:**
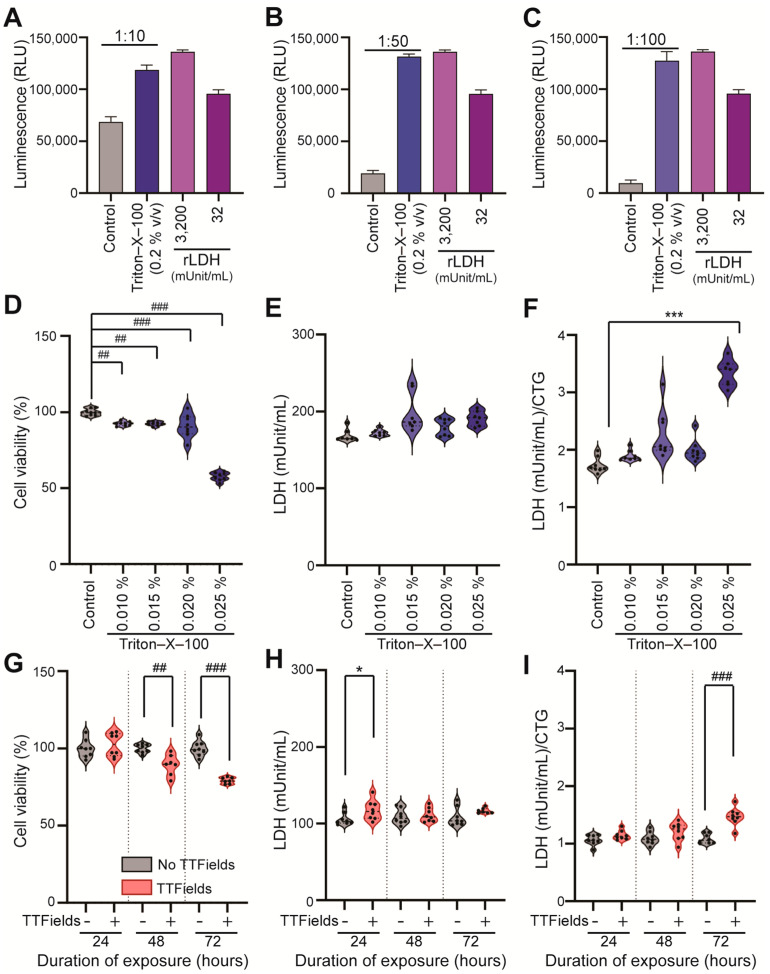
Optimized LDH release assay detected TTFields-induced human GBM cell membrane permeabilization in a time-dependent manner, with high sensitivity. (**A**–**C**) The sensitivity of the LDH assay was evaluated by optimizing the dilution of the cell culture media samples after a 10 min exposure of the cells to Triton-X-100 (0.2% *v*/*v*). Control (gray bar) represents the different dilutions of the media samples that were collected from the cells that had previously not been exposed to Triton-X-100: (**A**) 1:10, (**B**) 1:50, and (**C**) 1:100. The indigo bar in (**A**–**C**) represents the media samples that had been exposed to Triton-X-100 (0.2% *v*/*v*). The two purple bars in (**A**–**C**) show the luminescence generated by the recombinant LDH reference standard diluted to 3200 mUnit/mL (light purple) and 32 mUnit/mL (dark purple), for comparison purposes. (**D**) Cell viability by CTG assay, (**E**) LDH release, and (**F**) normalized LDH/CTG ratio for cells unexposed to Triton-X-100 (control, gray) and exposed to Triton-X-100 for 10 min (0.01–0.025%, shades of indigo). (**G**) Cell viability by CTG assay, (**H**) LDH release, and (**I**) normalized LDH/CTG ratio for cells exposed to no-TTFields (gray) or exposed to TTFields (salmon) for 24–72 h. Three independent experiments were performed, and either a parametric test (#) or a non-parametric test (*) was applied for statistical analysis. * indicates *p* < 0.05, ## *p* < 0.01, and ### or *** *p* < 0.001 compared to the no-TTFields or control (unexposed) group. CTG = CellTiter-Glo; rLDH = recombinant lactate dehydrogenase.

We optimized the LDH release assay to evaluate LDH enzyme release as a measurement of cell permeability in TTFields-exposed (1–4 V/cm, 200 kHz, 24–72 h duration) and -unexposed cells. We first exposed the cells to the positive control, Triton-X-100, at a concentration of 0.2% *v*/*v* for 10 min at 37 °C to standardize the sample dilution and optimize the sensitivity of the LDH assay in U87 cells. We then used different dilutions of the cell culture media (samples) to measure LDH release: 1:10, 1:50, and 1:100. We found that the assay remained sensitive even at 1:100 (see Figure 4A–C).

As a positive control, we exposed U87 cells to a Triton-X-100 solution at concentrations ranging from 0.010% to 0.025% (*v*/*v*) for 10 min at 37 °C and conducted CTG and LDH assays. The results indicated significant concentration-dependent differences in cell viability following exposure to Triton-X-100. Specifically, we observed the following results compared to the control (100 ± 2.25%) using a one-way ANOVA: 0.01% (*v*/*v*) Triton-X-100 (92.8 ± 1.45%, *p* = 0.002); 0.015% (*v*/*v*) Triton-X-100 (92.45 ± 1.32%, *p* = 0.002); 0.02% (*v*/*v*) Triton-X-100 (90.95 ± 7.65%, *p* = 0.0001); and 0.025% (*v*/*v*) Triton-X-100 (57.76 ± 2.72%), *p* < 0.0001 (Figure 4D). There was no significant difference in LDH release between the control and Triton-X-100 conditions (Figure 4E). However, when we compared the LDH/CTG ratio between the control condition (1.68 [1.65, 1.85]) and the 0.025% (*v*/*v*) Triton-X-100 condition (3.40 [3.14, 3.50]), there was a significant difference using the Kruskal–Wallis H test (*p* = 0.001) (Figure 4F).

We subsequently measured the viability of cells that had been exposed to TTFields or no-TTFields. The TTFields were applied at a strength of 1–4 V/cm and a frequency of 200 kHz for 24 to 72 h; we then measured cell viability via CTG and assessed bioluminescence using a plate reader. The results indicated significant time-dependent differences in cell viability, consistent with prior findings [19,25]. After 48 h of exposure to TTFields, the viability of the exposed cells was 89.47 ± 6.61% compared to 100 ± 2.76% for the control cells (*p* = 0.001, Student’s *t*-test). After 72 h, the viability was 79.27 ± 2.05% for the TTFields-exposed cells compared to 100 ± 4.95% for the controls (*p* < 0.0001, Student’s *t*-test) (Figure 4G). These data represent the results of three independent experiments.

The LDH assay results showed significant differences between TTFields and no-TTFields only at the 24 h duration (117.5 [107.1, 125.7] vs. 102.7 [100.7, 112.5] mUnit/mL, *p* = 0.028, TTFields vs. no-TTFields, Mann–Whitney U test) (Figure 4H). We also observed a significantly higher LDH/CTG ratio after 72 h (1.47 ± 0.15 vs. 1.08 ± 0.08, *p* < 0.0001, TTFields vs. no-TTFields, Student’s *t*-test) (Figure 4I). The data represent findings from three independent experiments.

### 3.4. Normalized LDH/CTG Ratio Detects GBM Cell Membrane Permeabilization Induced by the Combination of TTFields with Chemotherapy in Surviving Cells

Finally, we determined whether the CTG and LDH-CTG methods could detect an additive effect on cell membrane permeabilization after combined exposure to TTFields and carboplatin chemotherapy.

**Figure 5:**
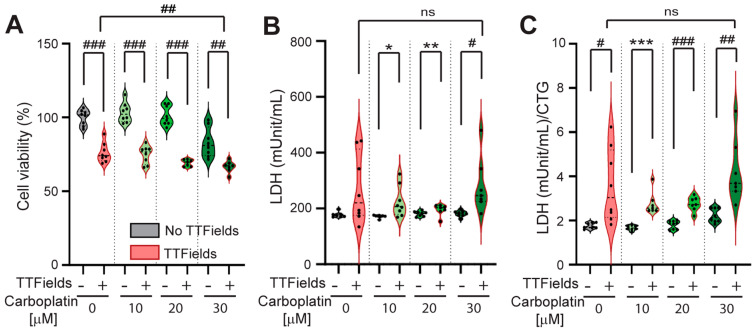
Normalized LDH/CTG ratio detected the GBM cell membrane permeabilization induced by the combination of TTFields with carboplatin chemotherapy in the surviving cells. (**A**) Cell viability was measured by CTG assay, (**B**) LDH release, and (**C**) normalized LDH/CTG in U87 cells exposed to carboplatin (0–30 µM) ± TTFields for 72 h. Two independent experiments were performed, and either a parametric test (#) or a non-parametric test (*) was applied for statistical analysis. # or * indicates *p* < 0.05, ## or ** *p* < 0.01, and ### or ***p < 0.001 compared to the no-TTFields or control (unexposed) group. CTG = CellTiter-Glo; LDH = lactate dehydrogenase; ns = not statistically significant.

The cells were exposed to TTFields (1–4 V/cm, 200 kHz, 24–72 h) and different concentrations of carboplatin (0, 10, 20, and 30 µM) that were lower than the 3-day carboplatin IC50 in these cells. After 72 h, we observed a significant reduction in the viability of cells exposed to the combination of TTFields and carboplatin at each carboplatin concentration compared to carboplatin alone. Figure 5A shows the results of the TTFields and no-TTFields conditions at each concentration (0 µM: 76.03 ± 6.62% vs. 100 ± 5.29%, *p* < 0.0001; 10 µM: 74.75 ± 6.06% vs. 103.2 ± 6.62%, *p* < 0.0001; 20 µM: 69.25 ± 2.29% vs. 101.3 ± 6.64% *p* < 0.0001; and 30 µM: 66.64 ± 3.89% vs. 82.98 ± 9.92%, *p* = 0.0013, Student’s *t*-test).

Conversely, after 72 h, we observed a significant increase in LDH release from the cells exposed to the combination of TTFields and carboplatin at each carboplatin concentration (except for 0 µM) compared to carboplatin alone. Figure 5B shows the results of the TTFields and no-TTFields conditions at these concentrations (10 µM: 207.3 [179.1, 275.9] vs. 173.6 [170.1, 174.0] mUnit/mL, *p* = 0.014, Mann–Whitney U-test; 20 µM: 246.5 [225.0, 342.5] vs. 182.9 [175.4, 188.9] mUnit/mL, *p* = 0.004, Mann–Whitney U-test; and 30 µM: 282.3 ± 100.1 vs. 181.8 ± 10.54 mUnit/mL, *p* = 0.014, Student’s *t*-test).

After 72 h, we observed a significant increase in the LDH/CTG ratio of the cells exposed to the combination of TTFields and carboplatin at each carboplatin concentration compared to carboplatin alone. Figure 5C shows the results of the TTFields and no-TTFields conditions at each concentration (0 µM: 3.57 ± 1.66 vs. 1.78 ± 0.13, *p* = 0.014, Student’s *t*-test; 10 µM: 2.56 [2.45, 2.91] vs. 1.66 [1.60, 1.77], *p* = 0.0006, Mann–Whitney U-test; 20 µM: 2.75 ± 0.33 vs. 1.80 ± 0.19, *p* < 0.0001, Student’s *t*-test; and 30 µM: 4.24 ± 1.44 vs. 2.22 ± 0.31, *p* = 0.002, Student’s *t*-test). CTG- and LDH/CTG-optimized methods were able to measure the cancer cell membrane permeabilization induced by the combination of TTFields and carboplatin.

Table 1 provides a comparative summary of the optimized methods on the basis of advantages, requirements, costs, and direct vs. indirect measurement capabilities.

## 4. Discussion

**Figure 6:**
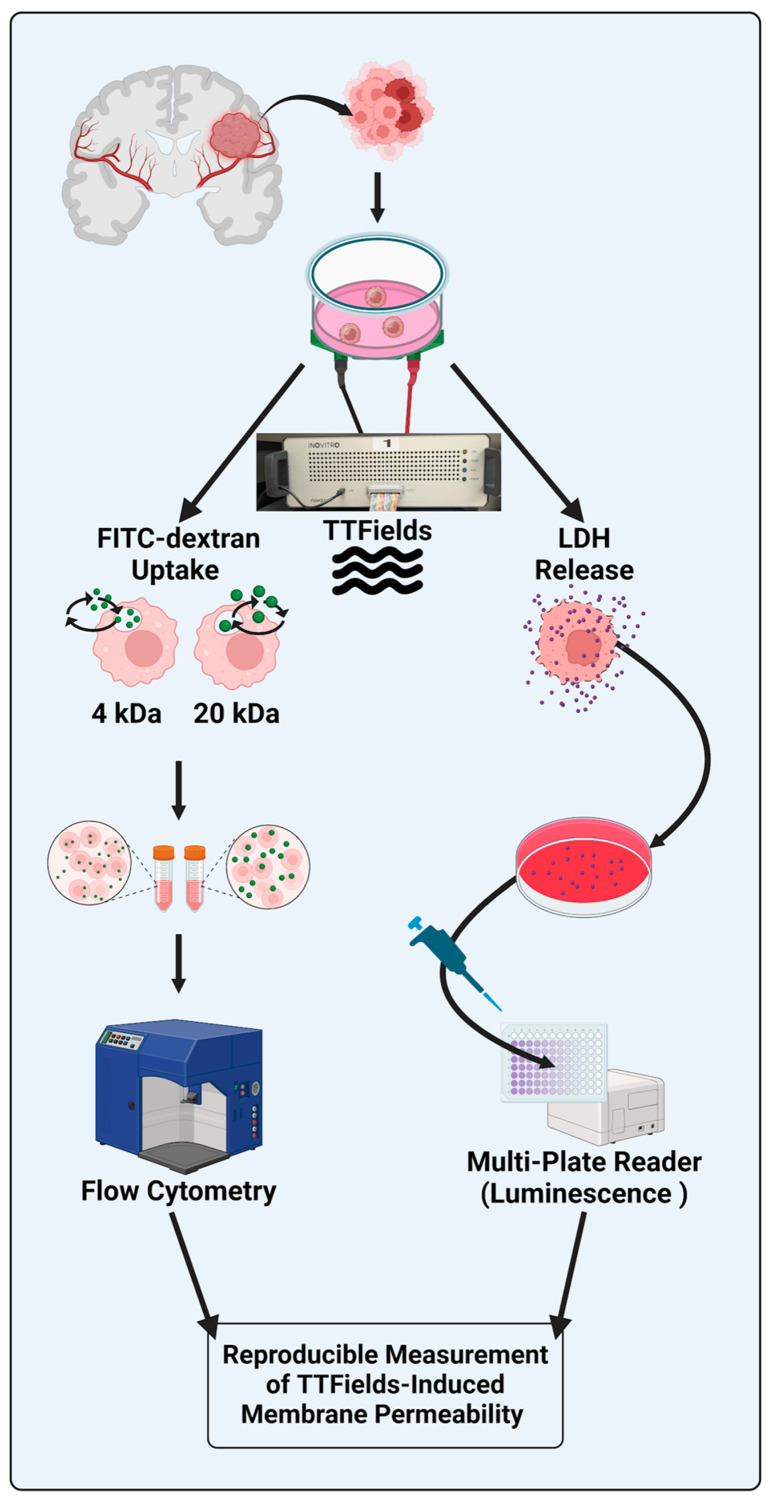
Graphical summary of the methods used in human GBM cells for evaluating TTFields-induced cell membrane permeabilization. Created with BioRender.com.

The cell membrane-disrupting properties of TTFields have been identified in human GBM cells in vitro [19], brain vascular endothelial cells in vitro [17,26], an orthotopic glioma murine model [17], and patients with GBM [18]. It is believed that this phenomenon is akin to that of reversible electroporation [20]. Given the emergence of this novel effect of TTFields, methods that accurately and reproducibly assess this mechanism are required. Previously reported methods (e.g., scanning electron microscopy and multi-well plate-based fluorescence readouts) are limited by the availability of necessary equipment and the inability to assess the permeabilizing effects on the cells that survive after the experiment. Here, we showed that FC and the LDH/CTG ratio can reproducibly quantify the cancer cell membrane-permeabilizing mechanism of TTFields. Figure 6 shows a graphical summary of the methods used in this study.

The reproducibility of the results of an experiment is key to increasing confidence in the data interpretation and ultimate conclusions; optimized methods and protocols play critical roles in achieving this goal [27,28]. In this study, we optimized both the FC-based (MFI) and LDH release-based (LDH/CTG ratio) methods using U87 cells grown at different times (different passage numbers and exposed to TTFields for 1–3 days). The results showed that both methods reproducibly demonstrated TTFields-induced membrane permeabilization in the cells, as evidenced by significant increases in the FITC-dextran MFI and LDH/CTG ratio in independent experiments after a 3-day exposure.

The sensitivity of the methods relies on their ability to be applied across various agents that target cancer cell membrane permeability. For example, Triton-X-100 is commonly used to permeabilize cell membranes. Nitsch and colleagues exposed renal non-cancer (RC-124) and cancer (786-O, Caki-1) cells to Triton-X-100 (dose range: 10^−5^% to 10^−2^%) for 5 min and found dose-dependent membrane permeabilization [29]. Cell membrane permeabilization was quantified using two approaches: (1) uptake of non-fluorescent fluorescein diacetate into the cells, metabolism and conversion into the fluorescent dye fluorescein, and readout by FC; and (2) cell incubation with fluorescein diacetate, followed by three washes (each involving 350× *g* centrifugation and resuspension in PBS), 0.001% Triton-X-100 exposure, sedimentation of cells (27× *g*), 20 min Triton-X-100 exposure, and analysis of cell-free supernatant via a fluorescent plate reader [29]. In this study, we showed that the permeabilization of the GBM cell membranes by a biophysical energy (i.e., TTFields) can be compared to the permeabilization induced by a surfactant detergent (i.e., Triton-X-100). Figure 3 shows that 1–3 days of 200 kHz TTFields exposure had a comparable effect on 4 and 20 kDa FITC-dextran uptake into human GBM cells to that of a 10 min 0.018% Triton-X-100 exposure and a 10 min 0.010–0.025% Triton-X-100 exposure, respectively. With respect to LDH, a recent study reported that LDH release is a suitable method for longitudinal monitoring and endpoint assessment of therapeutic efficacy in both cell line-derived xenografts and patient-derived explant cultures [30]. Further, the LDH/CTG ratio can detect the membrane permeability normalized to viable cells after exposure to cytotoxic chemotherapy (carboplatin), TTFields, or Triton-X-100. In this study, we validated that 1–3 days of 200 kHz TTFields exposure had a comparable normalized LDH release from human GBM cells (LDH/CTG ratio) to that of a 10 min 0.02–0.025% Triton-X-100 exposure. The ratio of absolute LDH to normalized CTG (LDH/CTG) for the TTFields and Triton-X-100 exposures was specific to the number of cells (U87 cells) and duration of exposure, which can vary from experiment to experiment.

In the past decade, novel methods have emerged for measuring cell membrane permeability in response to electrical pulses and electrical fields. For example, Sweeney et al. custom-made a device to apply pulsed electric fields to Chinese hamster ovary (CHO-K1) cells in culture; after staining the cells with propidium iodide, they used fluorescence microscopy to generate the electroporation threshold curves [31]. A follow-up study from the same authors used a microfluidic device and fluorescence microscopy of propidium iodide staining of CHO-K1 cells to quantify the trans-membrane transport behavior induced by single-pulse electric fields [32]. Computational modeling and simulations have also been used to quantify cell membrane permeability [33]. While these innovative approaches permit real-time monitoring of cell membrane permeabilization, they remain exploratory and are not yet widely adopted.

The methods used in this study are implementable in laboratories with access to a flow cytometer and multi-well plate reader and are not compatible with real-time monitoring. Although the media from experimental conditions can be sampled at various time points for the LDH quantification assay, the CTG cell viability assay results in cell death (i.e., a terminal readout). Thus, to determine temporal changes in the LDH/CTG ratio, which indicates the permeability of the membranes of the surviving cells after TTFields exposure, the number of samples required for this readout is limited by the number that can be assessed for cell viability. Filice and colleagues used scanning electrochemical microscopy and 3D finite element analysis simulations that matched the experimental and simulated results to correlate live cell viability with membrane permeability disruption induced by trivalent chromium [34]. Using readily available assays (i.e., LDH release and ATP-dependent cell viability determination), the LDH/CTG ratio used in this study provides another approach to correlating these aspects of cancer cell biology.

The size range of 4–20 kDa was chosen for the FITC-dextran probes because most chemotherapies and molecularly guided therapies used to treat patients with glioma (i.e., temozolomide [194.2 g/mol], procarbazine [221.3 g/mol], lomustine [233.7 g/mol], carboplatin [371.2 g/mol], vorasidenib [414.7 g/mol], irinotecan [586.7 g/mol], and vincristine [825.0 g/mol]) are smaller in size. We used 2 mL of FITC-dextran probes for the previously reported plate-based readout method [19], per GCS (1.0 mg/mL of 4 or 20 kDa). The quantity used in the current study was smaller, in terms of one-fourth the volume (0.5 mL) of the FITC-dextran probes used per GCS (0.72 mg/mL each). Therefore, staining cells with FITC-dextran for the FC-based readout in this study provided a cost-effective advantage (5.6-fold less for the 4 kDa and 20 kDa FITC-dextran probes) compared to our prior method [19]. This was due to the ability, in this study, to detach the cells from the GCS, wash them, and reconstitute them in a smaller volume prior to incubating them with the FITC-dextran probe.

In regard to the clinical application of TTFields in patients with glioblastoma and thoracic malignancies, this alternating electric fields therapy has been identified to exert its anti-cancer effects through various mechanisms including anti-mitotic effects, cellular disruption (e.g., membrane pore formation, disruption of the cytoskeletal network, and changes in cellular ionic content), DNA damage and inhibition of DNA damage repair, and immunogenic cell death among others [16]. Although TTFields monotherapy was shown to have a non-inferior survival benefit compared to physicians’ best choice of chemotherapy in patients with recurrent GBM [1], it is combined with standard systemic therapies in patients with newly diagnosed GBM [2,3], malignant pleural mesothelioma [7], and non-small cell lung cancer [8]. TTFields have been shown to improve the efficacy of standard anti-GBM chemotherapies in glioblastoma cells [35] and glioblastoma stem-like cells [36]. TTFields may enhance the efficacy of these standard systemic therapies by permitting their increased access to the cancer cells, which would need to be confirmed in vivo through appropriate methods (e.g., mass spectrometry).

The demonstrable survival benefit of the addition of TTFields therapy to standard systemic therapy for multiple cancer types has been demonstrated via clinical trials, and it has been demonstrated in the real-world setting in patients with recurrent [4] and newly diagnosed [5,6] GBM. Although tumor heterogeneity is a concern for eventual resistance to systemic anti-cancer therapies (e.g., pharmaceuticals and cell-based therapies), it may be the case that this is not as grave a concern in regard to the local delivery of TTFields therapy. For example, compared to standard temozolomide chemotherapy alone, TTFields combined with temozolomide prolonged overall survival in patients with newly diagnosed GBM, regardless of O^6^-methylguanine DNA methyltransferase (MGMT) promoter methylation status [2]. This is important to note because epigenetic silencing of the MGMT promoter via methylation impairs the repair of DNA damage caused by temozolomide, thereby conferring improved survival outcomes in patients with glioblastoma compared to those patients without MGMT promoter methylation [37]. Thus, TTFields appear to benefit patients with GBM despite the heterogeneity of the genetic and epigenetic patient and tumor profiles.

LDH is released into the bloodstream in other diseases that cause cell injury (e.g., rhabdomyolysis [38] and myocardial necrosis resulting from infarction [39]). However, there are conflicting results about the utility of LDH as a blood biomarker in patients with GBM [40,41], which may be due to the blood–brain barrier. In the in vitro system used in this study (24–72 h of ±TTFields exposure to human GBM cells in 2D monoculture), the likelihood of necrosis was low. This is supported by the fact that despite a significantly reduced cell viability in cells exposed to TTFields + 30 μM carboplatin, compared to TTFields alone (Figure 5A), there was no significant difference in either the LDH release (Figure 5B) or the normalized LDH/CTG ratio (Figure 5C). This suggests that the LDH/CTG ratio is a more specific measure to assess the permeabilizing effects of TTFields on cancer cell membranes, particularly in in vitro studies.

This study had a few limitations. Initially, twice the number of GCSs were required per sample for the FC-based readouts than in the previously reported plate-based readouts because at least 10,000 live cell counts were required for reliable results. However, through the optimization process, the number of GCSs per sample was reduced to one. Although doublets and dead cells were excluded from the FC-based analysis using the consistent gating strategy guided by a live/dead cell dye, there is a chance that some dead cells or debris were included in the FC counting solution, thereby extending the time of the overall procedure. Finally, this study evaluated the methods in a single human GBM cell line as a framework to demonstrate the necessary steps for pre-testing and optimization. This process can be generalizable to other cancer cell lines and TTFields experimental conditions (e.g., cell seeding density or duration of exposure), but the determination of the optimal concentrations of the FITC-dextran probes and detergent-based positive control (Triton-X-100) in various cell lines was outside the scope of this study. Optimizing and testing the methodologic framework presented here in different in vitro systems (based on varying cell types, drugs, TTFields parameters, etc.) would improve the generalizability and strengthen the conclusions of future studies.

Regarding future directions, in addition to the macromolecular tracer (i.e., FITC-dextran) based approach using FC and the LDH/CTG ratio reported in this study, future studies may evaluate other methods of quantifying cancer cell membrane permeability in response to TTFields (e.g., impedance-based assays [42]). For example, Salvador and colleagues measured the effects of TTFields on a 3D co-culture model of the blood–brain barrier composed of human primary microvascular brain endothelial cells and immortalized pericytes using transendothelial electric resistance measurements [26]. Future investigations are needed to validate these methods using surrogate assays (e.g., with drugs or particles of different sizes, whose uptake is monitored via microscopy). Furthermore, these methods could be employed to evaluate the therapeutic effects of TTFields combined with drugs that do not readily cross the BBB to evaluate for additive or synergistic effects.

## 5. Conclusions

We optimized and validated two cost-effective methods that reproducibly quantify the human GBM cancer cell membrane permeabilization induced by TTFields. The findings, which were based on two independent methods (i.e., FC and LDH release assays), reproducibly demonstrated TTFields-induced cell membrane permeabilization in cancer cells that remained alive after TTFields exposure. These methods were useful for time-dependent evaluations of the TTFields-induced cell membrane permeabilization effect. We also showed that these methods detected the effects of an established cell membrane permeabilizer, Triton-X-100, as a positive control, and detected the membrane-permeabilizing effects of chemotherapy combined with TTFields in surviving cancer cells. Considering the comparable effects of TTFields and Triton-X-100, these methods may be useful for evaluating membrane permeability in other experimental and pathological conditions.

## Figures and Tables

**Table 1:** Comparative summary of the optimized methods based on advantages, requirements, costs, and direct vs. indirect measurement capability.

Method	Advantages	Requirements	Cost	Direct vs. Indirect Measurement
FC	Sensitive Requires a lower concentration of probes than plate-based readout methods	FC machine and software for data analysis	Less expensive than the plate-based readout method (5.6-fold less for 4 kDa and 20 kDa FITC-dextran probes)	Indirect measurement of size-based probe uptake into the cells
LDH release	Very sensitive Requires a very low volume (10 µL) of sample or media High throughput and suitable for temporal measurements Samples can be frozen for later analysis	Plate reader or luminometer Reagent stored at −80 °C	Relatively costly	Indirect measurement of cell membrane permeability via LDH release
Cell viability (CTG)	Very sensitive Fast assay Easy to use Scalable to any plate format	Opaque white culture plates Plate reader or luminometer	Relatively costly, particularly for 6- or 12-well plates with larger well surface areas	Direct ATP-based measurement of the number of viable cells

## Data Availability

The data presented in this study are available upon reasonable request from the corresponding author.

## Supplementary Materials

The following supporting information can be downloaded at https://www.mdpi.com/article/10.3390/mps8010010/s1: Figure S1: Triton-X-100 (positive control) concentration testing in human U87 GBM cells (15 min exposure) prior to FC-based readout experiments of 4 kDa and 20 kDa FITC-dextran probe uptake. U87 cells were exposed to different concentrations of Triton-X-100, as indicated (0–0.2% [*v*/*v* in water]), for 15 min in cell culture media. Brightfield images were captured with a 10× objective lens.

## Abbreviations

CTG	CellTiter-Glo^®^
DMEM	Dulbecco’s modified Eagle’s medium
FBS	fetal bovine serum
FC	flow cytometry
FITC	fluorescein isothiocyanate
GBM	glioblastoma
GCS	glass coverslip
LDH	lactate dehydrogenase
MFI	mean fluorescence intensity
MGMT	O^6^-methylguanine DNA methyltransferase
TTFields	tumor treating fields
