# Supplementary material for: Optimized Methods to Quantify Tumor Treating Fields (TTFields)-Induced Permeabilization of Glioblastoma Cell Membranes
Source: Methods Protoc. 2025 Jan 22;8(1):10. doi: 10.3390/mps8010010 (PMC11858626; doi:10.3390/mps8010010)
Supplement: Supplementary file 1 [file mps-08-00010-s001.zip › mps-3395135-supplementary.pdf]

Supplementary File

Optimized Methods to Quantify Tumor Treating Fields (TTFields)-Induced Permeabilization of Glioblastoma Cell Membranes

Melisa Martinez-Paniagua, PhD, Sabbir Khan, PhD, Nikita W. Henning, BSc, Sri Vaishnavi Konagalla, Chirag B. Patel, MD, PhD

Supplementary Figure S1.

Triton-X-100 (positive control) concentration testing in human U87 glioblastoma cells (15-minute exposure) prior to flow cytometry-based read-out experiments of 4 kDa and 20 kDa fluorescein isothiocyanate (FITC)-dextran probe uptake.

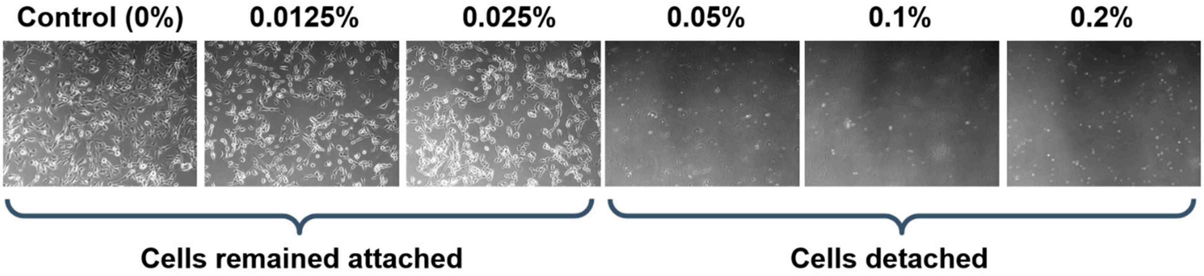

U87 cells were exposed to different concentrations of Triton-X-100, as indicated (0%-0.2% [v/v in water]), for 15 minutes in cell culture media. Brightfield images were captured with a 10× objective lens.
